# Significance of quantitative analyses of the impact of heterogeneity in mitochondrial content and shape on cell differentiation

**DOI:** 10.1098/rsob.230279

**Published:** 2024-01-17

**Authors:** Swati Agarwala, Sukhamoy Dhabal, Kasturi Mitra

**Affiliations:** ^1^ Department of Biology, Ashoka University, Delhi (NCR), India; ^2^ Department of Genetics, University of Alabama at Birmingham, Birmingham, AL, USA

**Keywords:** mitochondrial heterogeneity, mitochondrial shape, mitochondrial content, cell proliferation, cell differentiation, stem cells

## Abstract

Mitochondria, classically known as the powerhouse of cells, are unique double membrane-bound multifaceted organelles carrying a genome. Mitochondrial content varies between cell types and precisely doubles within cells during each proliferating cycle. Mitochondrial content also increases to a variable degree during cell differentiation triggered after exit from the proliferating cycle. The mitochondrial content is primarily maintained by the regulation of mitochondrial biogenesis, while damaged mitochondria are eliminated from the cells by mitophagy. In any cell with a given mitochondrial content, the steady-state mitochondrial number and shape are determined by a balance between mitochondrial fission and fusion processes. The increase in mitochondrial content and alteration in mitochondrial fission and fusion are causatively linked with the process of differentiation. Here, we critically review the quantitative aspects in the detection methods of mitochondrial content and shape. Thereafter, we quantitatively link these mitochondrial properties in differentiating cells and highlight the implications of such quantitative link on stem cell functionality. Finally, we discuss an example of cell size regulation predicted from quantitative analysis of mitochondrial shape and content. To highlight the significance of quantitative analyses of these mitochondrial properties, we propose three independent rationale based hypotheses and the relevant experimental designs to test them.

## Graphical abstract

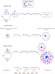

## 1. Introduction

Over the course of evolution, the prokaryote-turned-cellular-organelles, mitochondria, have been integrated into the functionality of the majority of the eukaryotic cells, almost in a customized way. Today we know mitochondria as multifunctional organelles with marked heterogeneity at different levels, which has been covered extensively in the reviews cited below in this and the following sections. Quantitative knowledge of the heterogeneity of various mitochondrial properties is necessary for understanding the critical nuances of mitochondrial functions and dysfunctions. In this review, we provide a necessary overview of the organelle and critically evaluate the current understanding of the quantitative aspects of certain mitochondrial properties in a certain cellular process towards generating multiple independent hypotheses.

Mitochondrial content varies between tissue and cell types, with differentiated aerobic cells at the maximum end and stem cells (undifferentiated) at the minimum end of the spectrum [1,2]. Mitochondria are also morphologically and structurally distinct between and within various tissue/cell types, and are also remodelled by various stimuli [3–6]. Within the same tissue type, mitochondrial shape and structure can vary based on local signalling [7–9]. Cells like neurons and myocytes exemplify striking intracellular heterogeneity of mitochondrial shape and function [10,11], while the same has been more recently documented for adipocytes [12]. Mitochondrial heterogeneity also exists between individual human subjects at the level of the circular DNA they carry (mt-DNA), which contributes to genetic variation [13]. Other than the classically described function of ATP production, mitochondria play critical roles in metabolic, redox and calcium homeostasis, which vary depending on the tissue type [5,14]. Mitochondria also serve as a pivot point of decision-making in the cell death process by controlling apoptosis [15]. Research over past decades has also revealed mitochondria as signalling organelles [16,17]. Therefore, a cell needs to maintain the right content, shape and kind of mitochondria that is achieved by regulating a mitochondrial life cycle. In such a life cycle, the mitochondrial content is controlled by the balance of mitochondrial biogenesis and mitophagy (mitochondrial clearance), while mitochondrial shape is controlled by a balance of mitochondrial fission and fusion processes [18].

Multifaceted involvement of mitochondria in various developmental and regenerative processes has gained wider attention. During normal development, pluripotent embryonic stem cells proliferate and differentiate into embryonic cell lineages that further differentiate into quiescent cells of various tissues [19]. Some of the differentiated cells in our adult body proliferate further to play critical roles in wound healing, immunity and integrity of various organs. Moreover, various adult tissues harbour adult stem cells that self-renew, proliferate and differentiate to replenish lineage specific dying cells. A self-renewing or proliferating cell has to exit the proliferating cycle to enter differentiation, which has to be regulated in a timely fashion. Aberrations in cell proliferation and differentiation can lead to various developmental, neurodegenerative and age-related disorders as well as cancer. Therefore, it is key to understand the fundamental concepts of mitochondrial involvement in cell proliferation and differentiation of stem cells and their progenies.

Here, we focus on two properties of mitochondria, namely mitochondrial content and shape, to highlight the significance of quantitative analyses of mitochondria. First, we critically review and evaluate the advances in the quantitative measurement of mitochondrial content and shape, particularly in proliferating or differentiating stem cells and their progenies. Thereafter, we generate three independent hypotheses for the role of mitochondrial content and shape regulation towards understanding the distinction of these mitochondrial properties between proliferating and differentiated cells. We also propose approaches of testing these independent hypotheses with quantitative analyses of mitochondria. Finally, we discuss an example of a quantitative link between mitochondrial content and shape through cell size scaling, which we hypothesize to be existing during cell proliferation and differentiation of stem cells and their progenies.

## 2. Quantitative aspect of heterogeneity of mitochondrial content in proliferating versus differentiated cells

Mitochondria are double membrane bound organelles with at least one mt-DNA nucleoid. The mt-DNA codes for 13 protein coding genes that are transcribed and translated inside the mitochondrial matrix with the help of tRNAs and rRNAs coded by the mt-DNA [13]. All the rest of the approximately 1100 mitochondrial proteins are coded by the nuclear DNA. Cells and tissue types vary in bioenergetics and metabolic abilities. Classically, mitochondria were studied primarily from aerobic differentiated tissues with higher mitochondrial content, like heart, liver and muscle. The tissue and cell type specific differences of mitochondria are largely maintained by distinct mitochondrial proteome (and phospho-proteome) and a varied mtDNA content between differentiated tissues [1,20,21]. For example, the respiratory chain complex IV, which consumes oxygen to generate ATP (oxidative phosphorylation), exhibits tissue specific isoforms [20].

The stem cells, harbour only few mitochondria that are specialized to maintain stem cell functionality (table 2). Various cells respond to increased metabolic demand and stresses by increasing their mitochondrial content that is driven by master regulators of mitochondrial biogenesis, like PGC1-α, Myc and mTOR [22–24]. To meet the demand of pathophysiologic situations, such as diabetes, heart failure or T-cell activation, mitochondria can also be remodelled during biogenesis [25–27]. The regulation of amount and kind of mitochondria also involves the process of mitophagy that removes mitochondria in a selective or non-selective manner [28]. Therefore, the make-up of the resultant mitochondrial content in any differentiated or proliferating cell is a function of mitochondrial biogenesis and mitophagy.

**Table 2.** Measurement of mitochondrial content in stem cells.

tissue/cell type	methods used	parameter measured	quantitative/semi-quantitative/ non-quantitative	molecular analyses- biogenesis or mitophagy/autophagy	fold change with differentiation	citation
neural stem cells/mice	qPCR; transmission electron microscopy imaging	complex V+ mitochondria; nuclear DNA content; mitochondria volume; gene regulation	quantitative or semi-quantitative	not tested	not tested	[79]
breast cancer stem-like cells	flow cytometry with MitoTracker	MitoTracker fluorescence	semi-quantitative	not tested	not tested	[80]
pluripotent stem cells, HEK293/human	biochemical analyses	citrate synthase activity normalized by total protein	semi-quantitative	not tested	no change	[81]
spermatogonia stem cells	flow cytometric analyses with MitoTracker; RT-PCR	mtDNA; fluorescence intensity	semi-quantitative	not tested	not tested	[82]
skin mesenchymal stem cells/mouse	imaging, biochemical analyses	CS activity; mtDNA	semi-quantitative	biogenesis and mitophagy	3× (adipogenesis); 2× (osteogenesis)	[83]
embryonic stem cells	real-time PCR analysis	mtDNA content per cell	semi-quantitative	biogenesis	∼3.5×	[2]
adipogenic differentiation of human mesenchymal stem cells	flow cytometry measurement of MitoTracker; immunoblotting	mean fluorescence intensity; TOM20	semi-quantitative	biogenesis	∼2×	[84]
embryonic stem cells/human	fluorescence microscopy of using Mitotracker; real-time PCR	mtDNA copy number	quantitative	biogenesis	∼2×	[85]
satellite cells isolated from post-mortem skeletal muscle stem cells	3D imaging; fluorescence microscopy; immunoblotting	TOM22; mtDNA content	semi-quantitative	not tested	not tested	[86]
human ESCs and iPSCs. H1- and H9-derived fibroblasts	qPCR	mtDNA copy number	quantitative	biogenesis	∼5×	[87]
neural progenitor cells	real-time PCR	mtDNA copy number calculated by normalizing to nuclear genomes- per cell basis	semi-quantitative	biogenesis	∼4×	[72]
human embryonic stem cells (hESCs) and hESC-derived neural stem cells	fluoroscence microscopy using MitoTracker Red and calcein-AM; qPCR	mtDNA, mit. volume, content	quantitative	biogenesis	∼0.5	[88]
undifferentiated hESC/hiPSC cells and adult dermal skin fibroblasts	qPCR	relative amounts of the mitochondrial gene(s) ND1, ND5, and MT-CYB	not quantitative	biogenesis	∼200×	[89]

Mitochondria consist of all the major biological macromolecules, namely protein, lipid, DNA and RNA. Therefore, ideal analyses of mitochondrial content include assessment of the mitochondrial protein, lipid, mt-DNA (and RNA). The methods used are based on biochemical analyses, fluorescent and electron microscopy, polymerase chain reaction (PCR), flow cytometry, genomics and proteomics. These methods can quantitatively or semi-quantitatively measure parameters like mitochondrial protein levels, mitochondrial enzyme activities, mt-DNA levels, mitochondrial number, mitochondrial lipid content and gene expression. Employing a single method to estimate mitochondrial content could lead to errors, as reflected in the following examples. Use of mitotracker dyes in haematopoietic stem cells can be erroneous due to the ability of cells to actively extrude the dye [29]. Also, mitochondrial content measured by transmission electron microscopy in human subjects did not correlate with mt-DNA content, while it strongly correlated with cardiolipin content and citrate synthase activity [30]. A mass spectrometry-based approach for quantitative comparison of mitochondrial content across four mouse tissues used a novel parameter named ‘mitochondrial enrichment factor' (MEF) [31]. The authors, based on their observations, caution that levels or activity of no single mitochondrial protein (ex: citrate synthase) can be reflective of mitochondrial content as opposed to the standard practice. Also, mitochondrial number may not reflect mitochondrial content in cases where the majority of the mitochondrial content are in large hyperfused mitochondrial networks [32,33]. Furthermore, an increase in mitochondrial content in response to mitochondrial stress may not always lead to increases in mitochondrial function [34]. Therefore, accurate understanding of the functional significance of alteration of mitochondrial content is best possible with multiparametric analyses. Here, we have listed the different methods used to assess mitochondrial content and its regulation by mitochondrial biogenesis and/or mitophagy in various tissue/cell types, including stem cells (tables 1 and 2). It is noteworthy that the metrics obtained using different methodologies and detection platforms cannot be considered as absolute measures and thus cannot be compared between studies.

**Table 1.** Measurement of mitochondrial content in differentiated cells.

tissue/cell type	methods used	parameter measured	quantitative/semi-quantitative/descriptive	molecular analyses- biogenesis or mitophagy/autophagy	citation
*Drosophila* axons	fluorescence microscopy of mitochondrial marker	mitochondrial number	quantitative	biogenesis	[35]
*Drosophila* jurkat/human and Kc167	flow cytometry of MitoTracker	mitoTracker fluorescence intensity	semi-quantitative	mitophagy	[36]
*C. elegans* germline nuclei	live imaging microscopy; multiplex droplet digital PCR	per cent mtDNA frequency; mitochondrial content	quantitative	biogenesis	[37]
*Saccharomyces cerevisiae*	fluorescence microscopy of mitochondrial marker	mitochondrial volume derived from mitochondrial length	quantitative	not tested	[38]
yeast strain wild-type and TPK3 cells-	dual-beam spectrophotometer	mitochondrial cytochromes-aa3, b, and cc1	semi-quantitative	biogenesis	[39]
mouse cortical neurons	fluorescence microscopy of MitoTracker and gene expression analyses	mitoTracker fluorescence intensity, fold change in expression of multiple mitochondrial markers; mtDNA	semi-quantitative	biogenesis	[40]
mouse hepatocytes	electron microscopy	number, size, volume of mitochondria per hepatocyte	quantitative	not tested	[41]
mouse skeletal muscle	electron microscopy and RT-PCR	mitochondrial number and mtDNA:nDNA	quantitative or semi-quantitative	biogenesis	[34]
cohen rats fibroblast	fluorescence microscopy of MitoTracker and gene expression analyses	fluorescence intensity, fold change in expression of multiple mitochondrial markers; mtDNA	semi-quantitative	biogenesis	[42]
mouse hepatocyte	flow cytometry of MitoTracker	MitoTracker fluorescence intensity	semi-quantitative	biogenesis	[43]
mouse hippocampal astrocytes	fluorescence microscopy of MitoTracker	MitoTracker fluorescence intensity	semi-quantitative	not tested	[44]
mouse astrocytes	fluorescence microscopy of MitoTracker; RT-PCR	mitochondrial volume normalized by astrocyte volume, fold change of mRNA	quantitative or semi-quantitative	biogenesis	[45]
mouse NIH/3T3 cells, myofibroblasts compared to native fibroblast	microscopy using fluorescent dyes; biochemical analyses	mean intensity; number of polarized mitochondria; mtDNA; mt. proteins	quantitative or semi-quantitative	not tested	[46]
rat optic nerve head astrocytes	microscopy of MitoTracker and biochemical	volume density; mt. number per area; mt. protein expression in terms of fold change	quantitative or semi-quantitative	biogenesis	[47]
rat testes, leidig cells	fluorescence microscopy of MitoTracker	fluorescence intensity	semi-quantitative	biogenesis	[48]
rat primary cortical astrocytes	microscopy, biochemical analyses; Western blotting	mitochondria mass; OXPHOS, % relative expression of mt. proteins	semi-quantitative	not tested	[49]
mouse synaptic boutons of axons; neurons	fluorescence microscopy of MitoTracker; immunogold staining	normalized to cell area, mitochondria length per length of axon; mean fluorescence (normalized to cell area); mitochondrial area	semi-quantitative	not tested	[50]
mouse diet-induced fatty liver in C57BL/6J/	biochemical analyses; Western blotting	CS activity; mt protein: tissue protein; TG content	semi-quantitative	not tested	[51]
mouse astrocytes cultured cells	fluorescence microscopy of MitoTracker	fluorescence intensity	semi-quantitative	not tested	[52]
rat Myc null fibroblasts	flow cytometry using MitoTracker and NAO	fluorescence intensity	semi-quantitative	biogenesis	[53]
mouse embryonic fibroblasts	flow cytometry analysis; fluorescence microscopy of using MitoTracker	fluorescence intensity	semi-quantitative	biogenesis and mitophagy	[54]
mouse embryonic fibroblasts	flow cytometry analysis; fluorescence microscopy of using MitoTracker	fluorescence intensity	semi-quantitative	autophagy	[55]
rhesus monkey presynaptic boutons	electron microscopy	mitochondrial number	quantitative	not tested	[56]
human skeletal muscle	transmission electron microscopy micrographs; biochemical analyses	mitochondrial volume and profile; CS and COX activity	quantitative or semi-quantitative	not tested	[57]
human: female skeletal muscle cross sections: type II fibre	immunohistochemical analyses	COXIV protein intensity	semi-quantitative	not tested	[58]
human post-mortem brain sections of OXPHOS deficient PD astrocytes	imaging mass cytometry	mitochondrial mass per astrocytic area/CIV area; relative expression of OXPHOS proteins normalization to Z scores; pixel intensity	semi-quantitative	not tested	[59]
human myotubes	biochemical analyses	mitochondrial yield per total cellular protein, NAO staining for cardiolipin, and level of mitochondrial markers	semi-quantitative	not applicable	[60]
human skeletal muscle biopsies	2D TEM imaging and Western blotting	citrate synthase activity, cardiolipin content, mitochondrial DNA content (mtDNA), complex I–V protein, and complex I–IV	semi-quantitative	not tested	[30]
human skeletal muscle cells (vastus lateralis muscle biopsies)	biochemical analyses; Western blotting	cytochrome c oxidase (COX) activity; fold change of biogenesis proteins and fission/fusion proteins	semi-quantitative	biogenesis	[61]
human striated muscle	3D microscopy based image rendering; biochemical analyses;	mitochondrial volume as a per cent of total muscle volume; oxidative demand	semi-quantitative	not tested	[62]
human vastus lateralis muscles	PCR for mtDNA CS activity;	mtDNA; CS activity	semi-quantitative	biogenesis	[63]
human males whole-muscle level of leg and arm muscles	transmission electron microscopy; biochemical analyses	mitochondrial volume fraction per volume of myofiber; HAD and CS activity; total mit. content in IMF and SS regions	semi-quantitative	not tested	[64]
human vastus lateralis single muscle fibres	imaging, combined with 3D reconstruction	mitochondrial volume correlated with potential biomarkers: respirometry, enzymatic activities, protein content	quantitative or semi-quantitative	not tested	[65]
human non-small cell lung cancer cells	staining with MitoTracker green	percentage of mitochondria mass	semi-quantitative	biogenesis	[66]
human HEK293 cells and primary dermal fibroblasts	FACS analysis; fluorescence microscopy of using MitoTracker	MTC02 staining analysis	semi-quantitative	mitophagy	[67]
HeLa cells	flow cytometry with MitoTracker	MitoTracker fluorescence	descriptive	not tested	[68]
HeLa cells	fluorescence microscopy of MitoTracker	MitoTracker fluorescence intensity	semi-quantitative	not tested	[69]
cultured muscle tissues	Western blotting; biochemical analyses	VDAC protein content for normalization	semi-quantitative	biogenesis	[70]
C2C12 myoblasts	qPCR	mt-DNA copy number	quantitative	biogenesis	[71]
neural progenitor cells	real-time PCR	mt-DNA copy number calculated by normalizing to nuclear genomes per cell basis	semi-quantitative	biogenesis	[72]
human neonatal foreskin HFF1 fibroblasts	FACS based fluorescence analysis using MitoTracker and NAO	fluorescence intensity	semi-quantitative	biogenesis and mitophagy	[73]
rat hepatocytes and HeLa cells	flowcytometry and microscopy of MitoTracker and biochemical	fluorescence intensity, number of mitochondria and citrate synthase activity	semi-quantitative	biogenesis	[74]
human and mice Tsc2-deficient neurons	flow cytometry-based assay	mitochondrial mass and number per segment; biogenesis	quantitative or semi-quantitative	biogenesis and mitophagy	[75]
mitochondria from liver, heart, kidney and brain	quantitative LC-MS	quantitative protein content	quantitative	not tested	[1]
brain, heart, liver, both kidneys and skeletal muscle	enzymatic activity; slot-blot method	mtDNA:nDNA; cytochrome c oxidase (COX) and citrate synthase (CS) specific activity per mtDNA:nDNA;	semi-quantitative	biogenesis	[76]
liver, quadriceps, heart, brain, and brown adipose	proteomic comparison using biochemical analyses	ATP synthase (complex V, or CV) component proteins	semi-quantitative	not tested	[77]
brown adipose tissue, heart, kidney, and liver	label-free, quantitative nLC-MS/MS.	sample specific mitochondrial enrichment factor (MEF)	quantitative	not tested	[31]
mouse myoblast cells, *C.elegans* touch receptor neurons, *Drosophila* heart and indirect, flight muscle, and mouse skeletal muscle	fluorescence microscopy of mitochondrial marker	mitochondrial marker positive pixels	semi-quantitative	mitophagy	[78]

**Figure 1.**
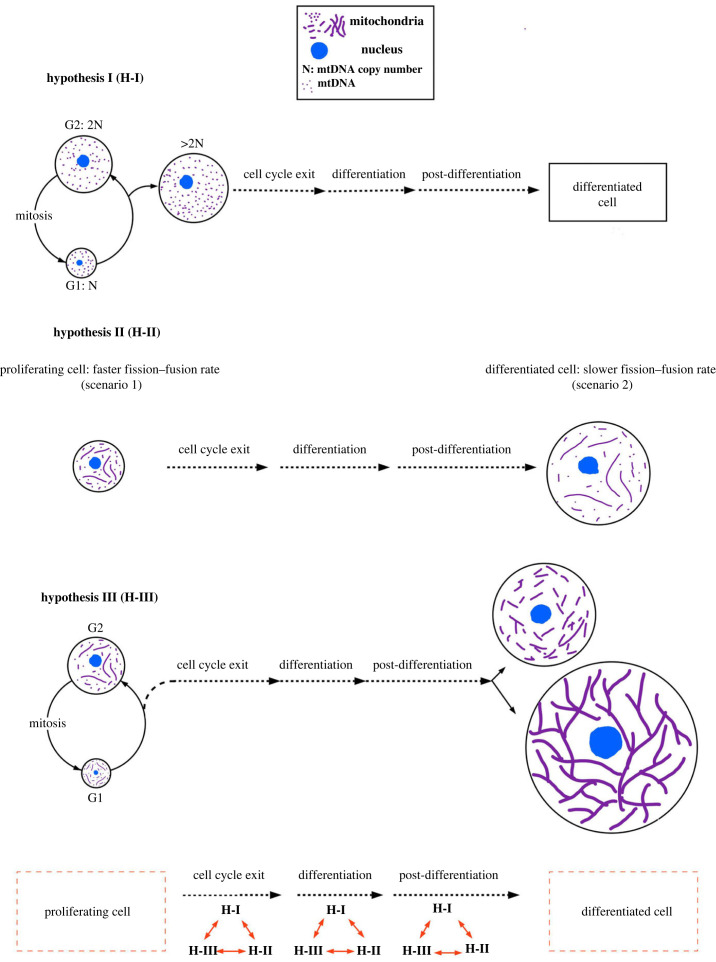
Three independent hypotheses are postulated on mitochondrial shape and content during cell differentiation, as proliferating cells undergo cell cycle exit, active differentiation and post differentiation. Hypothesis I, on the dependence of differentiation on mtDNA content, states: while a self-renewing/proliferating cell maintains strict control on a twofold increase in the mt-DNA copy number, a greater than twofold increase in mt-DNA copy number is sensed by the stem cell as a trigger for differentiation. Hypothesis II, on the differences of rates of mitochondrial fission–fusion between differentiated and proliferating counterparts, states: if scenario 1 represents a proliferating cell, scenario 2 with lower rates of mitochondrial fission and fusion events may represent the differentiated counterpart of that cell. Hypothesis III, on allometric scaling of mitochondria with cell size during differentiation, states: the reported quantitative allometric scaling of mitochondrial content/functionality with cell size, through alteration of mitochondrial shape, is established during differentiation of stem cells. The panel at the bottom indicates that the interaction of three independently hypothesized events (H-I, H-II, H-III) can potentially happen during any or all of the steps of cell cycle exit, active differentiation or post-differentiation.

During the process of cell proliferation, the net mitochondrial content can only increase by twofold with the doubling of cellular mass, thus maintaining the scaling of mitochondrial and other cellular contents. Mitochondria have been demonstrated to partition in proportion with the cytosolic volume in yeast and randomly between daughters in symmetrically partitioning mammalian cells [90,91]. Therefore, we reason that some active control of mitochondrial content is necessary during each round of cell proliferation. On the other hand, the cell differentiation process is associated with a dramatic increase in mitochondrial content [2,7,46,72,83,85]. In some cases, the increase in mitochondrial content has been causatively linked with entry into differentiation by employing genetic/genomic approaches *in vivo* or *in vitro* models [43,70,92–95]. Stem cells being a meaningful model to study differentiation, here we carefully surveyed the fold increase in mitochondrial content reported during stem cell differentiation. We consistently found differentiation of stem cells associates with a greater than twofold increase in mt-DNA copy number (that can exceed fivefold in some cases), across various studies on various cell lineages (table 2). Notably, comparison of other mitochondrial parameters measured did not reveal such a striking phenomenon in our survey. Consistently, measurement of certain mitochondrial parameters, excluding mt-DNA, has been found to be comparable between embryonic stem cells and their differentiated counterparts [81]. Therefore, we hypothesize that while a self-renewing/proliferating cell maintains a strict control on twofold increase in the mt-DNA copy number, a greater than twofold increase in particularly mt-DNA copy number is sensed by the stem cell as a trigger for differentiation (hypothesis I in figure 1). Evidence for such a conceptualization can be obtained with quantitative measurement of mt-DNA content in stem cells during the event of cell cycle exit for entering differentiation (see proposed hypothesis testing section).

## 3. Quantitative aspects of heterogeneity of mitochondrial shape and dynamics in proliferating versus differentiated cells

Subjective evaluation of mitochondrial shape within cells has revealed a wide spectrum, which can be discontinuous (discrete shapes) or continuous (different mitochondrial length, number or size). Examples of subjective descriptions of mitochondrial shape are clustered, aggregated, doughnut shaped, networked, tubular, hyperfused, net-like and fragmented [3,96,97]. An expanding body of literature also demonstrates that various pathophysiological stimuli alter mitochondrial shape in a given cell type [17,98,99]. Mitochondrial shape in any given cell is maintained by a balance in the dynamic opposing processes of mitochondrial fission and fusion that are collectively referred to as mitochondrial dynamics [97–99]. Mitochondrial fission refers to the process where a larger mitochondrion undergoes fission of their inner and outer membranes to form smaller mitochondria. Mitochondrial fission is driven by dynamin-related protein 1 (Drp1) that is recruited to the mitochondrial surface from the cytosol by bona fide mitochondrial proteins like mitochondrial fission factor 1 (MFF1) or fission protein 1 (Fis1). On the other hand, mitochondrial fusion refers to the process where two smaller mitochondria fuse both their membranes to form a larger mitochondrion. Transient fusion events happening between two mitochondria allow exchange of contents between them, without forming a larger mitochondrion. The outer mitochondrial fusion is driven by mitofusins (Mfn1/2) while the inner membrane fusion is primarily driven by optic atrophy 1 (Opa1). Influence of cytoskeletal elements and the endoplasmic reticulum on mitochondrial fission and fusion processes further reveals the complexity of how mitochondrial dynamics maintain the morphometric features of a given mitochondrial shape [100,101].

Quantitative understanding of mitochondrial shape along its wide morphometric spectrum is crucial for obtaining deeper insight into the structure–function relationship of mitochondria and how that impacts various cellular processes including differentiation of stem cells [97–99]. Analyses of mitochondrial morphometry obviously involves electron and fluorescence microscopy-based visualization and various image analyses tools. Tools to quantify mitochondrial morphometric parameters from micrographs, primarily of cells and tissues that are amenable to fluorescence microscopy, include multiple Image J plugins [102], MitoHacker [103] (for high-throughput two-dimensional analyses), MitoGraph (for high-resolution three-dimensional analyses) [104] and various customized algorithms [32,105,106]. A mitochondrial morphological complexity index of mouse hippocampus has been recently reported using a three-dimensional approach employing serial block face scanning electron microscopy [9]. MitoGraph output has been used in the MitoSinCe^2^ method to design quantitative metrics for assessing the contribution of mitochondrial fission/fusion on their structure–function in single cells [107]. An automated approach, named Mitometer, has been reported for identifying and quantifying mitochondrial fission and apparent fusion events by tracking dynamic mitochondria in cells [108]. Assessment of definitive fusion events of mitochondrial inner and outer membranes has been achieved by employing photoconvertible fluorescent probes in live cell pulse chase assays [107,109].

A clear distinction of mitochondrial shape between stem/progenitor cells and their differentiated counterparts has been reported in various lineages [96,110–114] (table 3). The majority of the studies on pluripotent stem cells (ESCs and iPSCs) have reported small punctate mitochondria with less matured cristae and bioenergetic functionality in the stem cells and elongated mitochondria in their differentiated counterparts [87,113,117,120,124,129]. On the other hand, elongated and fused mitochondria have been reported in adult stem cells of various lineages [8,96,112,124,129,134, 137–140], with some lineages maintaining punctate mitochondria [114]. Differentiation in stem cells has been shown to be supported by either mitochondrial fission or fusion, as investigated in different cell lineages [8,112,129,134,137–140]. The majority of such studies investigated the impact of mitochondrial shape at the extremes of the morphometric spectrum, largely leaving out the shapes in between the extremes. Precise quantification of mitochondrial network length revealed that maintenance of smaller network size (comprising of ≤ 40% of the total mitochondrial network size) supports stem cell gene expression profile in keratinocyte lineage, while almost complete fusion of mitochondrial network (hyperfusion) prevents it [128]. The smaller mitochondrial network size is reminiscent of the theoretical conceptualization of a quantitively defined ‘meso-fused’ mitochondrial structure, where the active fission rate dominates to maintain smaller mitochondrial network size [141].

**Table 3.** Mitochondrial shape in stem cells.

tissue/cell type	described mitochondrial shape	methods used	parameter measured	quantitative/semi-quantitative/descriptive	citation
human embryonic stem cells (hESC) mouse epiblast stem cells (EpiSC)	spherical	electron microscopy	subjective	descriptive	[115]
murine neural stem cells (NSCs)	elongated	confocal microscopy	mitochondrial length	quantitative	[8]
NSCs/hippocampal progenitors	mixed (globular and tubular)	electron microscopy	mitochondrial volume	quantitative	[116]
induced pluripotent stem cells (iPSCs)	spherical	electron microscopy	subjective	descriptive	[117]
rat bone marrow mesenchymal stem cells (BMSCs)	elongated, Interconnected	confocal microscopy	mitochondrial length	quantitative	[118]
human naive and primed ESCs	round in naive and elongated in primed ESCs	electron microscopy	mitochondrial length and width ratio	quantitative	[119]
ESC, iPSCs	spherical and round shaped	transmission electron microscopy	subjective	descriptive	[120]
human ESCs	both elongated and tubular	transmission electron microscopy	subjective	descriptive	[121]
mouse ESCs	globular	electron microscopy	mitochondrial length	quantitative	[122]
hESC, iPSCs	round	transmission electron microscopy	mitochondrial Diameter	quantitative	[87]
iPSCs and ESCs	immature-like morphology	TEM	subjective	descriptive	[123]
human PSCs	fragmented	confocal microscopy, TEM	subjective	descriptive	[81]
mouse ES and iPSCs	fragmented	electron microscopy	cristae length/mitochondrial area	quantitative	[124]
P19 embryonic carcinoma stem cells	small and round in P19SCs and filamentous in P19dCs	confocal and electron microscopy	mean area/ perimeter ratio (interconnectivity) and inverse circularity (elongation)	quantitative	[125]
human breast cancer stem cells (BCSCs)	branched mitochondrial network	confocal microscopy	subjective	descriptive	[126]
germline stem cells/*Drosophila*	tubular and branched	live confocal microscopy	subjective	descriptive	[127]
human neoplastic stem/progenitor cells	smaller, fused	confocal microscopy	mitochondrial Length	quantitative	[128]
mouse ESCs/differentiated cardiomyocytes	fragmented in ESCs and elongated in cardiomyocyte	electron microscopy, two-photon microscopy	mitochondrial length	quantitative	[129]
human ESC to differentiated gastric epithelial cells	small in hESC and large in differentiated cells	electron microscopy	subjective	descriptive	[130]
hESCs, iPSCs and differentiated cells	globular elongated elongated and globular	transmission electron microscope	subjective	descriptive	[131]
*Drosophila* neuroblasts	small and round	transmission electron microscope	mitochondrial length & surface	quantitative	[132]
*Drosophila* testis germline stem cells	punctate	transmission electron microscope	subjective	descriptive	[112]
subpopulations of tumor initiating cells	elongated or punctate mitochondria in different subpopulation	confocal microscopy	fission and Fusion and Diameter metrics	quantitative	[107]
quiescent or primed HSCs	punctate in quiescent HSCs and elongated in primed HSCs	confocal microscopy	subjective	descriptive	[133]
human neural cortical stem cells	large mitochondria	confocal microscopy	mitochondrial length	quantitative	[134]
bone marrow progenitors differentiated to to immature dendritic cells	short, tubular in progenitors and long tubular in dendritic cells	confocal microscopy	mitochondrial length	quantitative	[135]
young and aged and *Drosophila* germline stem cells	fragmented in aged and larger in young GSCs	confocal transmission electron microscopy	mitochondrial area and length	quantitative	[136]
reprogramming to induced pluripotent stem (iPS) cells	reprogramming intermediates contain fragmented mitochondria	TEM micrographs	mitochondrial length	quantitative	[137]

Dramatic alteration of mitochondrial shape happens both during the proliferating cycle of cells and in their differentiation process towards regulating such physiology [96,110,113,114]. In the light that mitochondrial physiology is distinct between proliferating and differentiated cells [142,143], the regulation and role of mitochondrial fission and fusion dynamics can also be potentially different between them. For instance, the estimated rate of mitochondrial fission and fusion dynamics in differentiated myocytes is of the order of days as opposed to seconds as observed in *in vitro* culture systems [144,145]. The higher rate of mitochondrial dynamics observed in differentiated cell line models or *ex-vivo* tissue models could be confounded by their proliferating status or isolation-stress induced change in mitochondrial shape [146]. Indeed, the rate of mitochondrial dynamics increases as quiescent mouse embryonic fibroblasts (alike quiescent differentiated cells) are induced to enter cell proliferation (unpublished results). Predictive theoretical considerations have revealed that mitochondrial fission and fusion rates can influence mitochondrial shape and dynamics independently [141]. Steady state mitochondrial shape (as observed in snapshot micrographs) can be determined by the ratio of fusion and fission rates, i.e. the number of mitochondrial fission or fusion events per unit time (as quantified from time lapse micrographs). In scenario 1, a cell with two fusion events/mito-element/minute and one fission event/mito-element/minute can potentially achieve and maintain a moderately fused network with fast mitochondrial dynamics. In scenario 2, a cell with two fusion events/mito-element/day and one fission event/mito-element/day would have identical steady state mitochondrial shape as in scenario 1 but exhibit much slower mitochondrial dynamics. We hypothesize that if scenario 1 represents a proliferating cell, scenario 2 with lower rates of mitochondrial fission and fusion events may represent the differentiated counterpart of that cell (hypothesis II in figure 1). Evidence for such a conceptualization can be obtained by measurement of absolute rates of mitochondrial fission and fusion events with advanced microscopy and image analyses techniques in proliferating cells and their differentiated counterparts (see proposed hypothesis testing section).

## 4. Quantitative analyses reveal link between mitochondrial content and shape in cell size regulation

The regulatory balances for mitochondrial shape (fission and fusion) and of mitochondrial content (biogenesis and mitophagy) have been proposed to be integrated through a mitochondrial life cycle [18]. Here, it is important to make the conceptual distinction between mitochondrial fission and division. Only mitochondrial division, and not fission, increases overall mitochondrial content when not balanced out by mitophagic clearance. On the other hand, fusion of two smaller mitochondria would not amount to mitochondrial biogenesis without a concomitant increase in the mass of the resultant fused mitochondria from that of the sum of the individual mitochondria. In the mitochondrial life cycle, mitochondrial fission and fusion counter each other, which is quantitatively captured in the inverse relationship of the metric measuring contribution of fission and fusion on mitochondrial shape [107]. The inverse relationship of mitochondrial fission and fusion metrics weakens below a certain level of the fusion metric. It remains to be quantitatively examined if and how modulation of mitochondrial biogenesis and/or mitophagic clearance may impact the aforementioned inverse relationship. However, mitophagic clearance is thought to operate on punctate mitochondria, and maintenance of mitochondrial tubules can prevent that [18,28]. Mitochondrial fission towards the end of a mitochondrial tubule can also be associated with mitophagic machineries in proliferating cells, whereas mitochondrial fission in the middle of a tubular mitochondria is associated with replicating mt-DNA [147]. However, replicating mt-DNA cannot be equated to mitochondrial biogenesis that results in an overall increase in mitochondrial content.

Genetic manipulation of mitochondrial fission or fusion machineries can dramatically impact mitochondrial content, biogenesis and mitophagy [7,144,145,148]. Extensive hyperfusion by greater than 90% reduction of the mitochondrial fission protein, Drp1, elevates mitochondrial number and gene expression of mitochondrial proteins and prevents stem cell maintenance in skin keratinocyte lineage [128]. Importantly, such a compensatory effect is not observed with fine-tuned repression of Drp1 that sustains stem cell gene expression in the same lineage [128]. Associated increases in mitochondrial fission and mitochondrial content have been causatively linked with the process of stem cell differentiation [45,71,110,113,114]. Excessive mitochondrial fission causes a bioenergetic crisis to potentially turn on the compensatory mechanism of mitochondrial biogenesis and push the cells out of the cell cycle and towards differentiation [98,144].

In general, differentiated cells are larger than their lineage specific stem cells [19]. Increase in cell size needs an overall boost in gene transcription and translation supported by increased anabolic activities, where biosynthetic and metabolic activities are scaled with cell size [149]. Overall, the gene transcription rate and protein levels are expected to be higher in proliferating cells with higher mitochondrial content and function, as suggested by both experimental and theoretical analyses [68,150]. The Largen gene, isolated in a screen to identify cell size regulatory genes, substantially increases mitochondrial content and boosts mitochondrial respiratory function by specifically augmenting the translation of mRNAs encoding mitochondrial proteins [151]. Similarly, the scaling of mitochondrial network size with cell size has been found to be linear in the growing yeast bud [38]. However, in a genetically engineered non-proliferating liver cell model that can grow in size, gene transcription for mitochondrial proteins was found to reduce in larger cells [152]. This observation on liver cells, which have higher ploidy than diploid cells, is consistent with the observations in other polyploid models [153–155]. Moreover, the quantitative allometric relationship of mitochondrial functionality (as measured by potentiometric dye incorporation) and cell size appears to be nonlinear, even in diploid proliferating cell models. There, the mitochondrial function is maximum in cells of intermediate size, beyond which it reduces with an increase in cell size [36]. It has been proposed that mitochondrial fission and fusion abilities change mitochondrial shape to compensate for reduced mitochondrial functionality, thus allowing effective mitochondrial function in larger cells. Such a conclusion is based on the result that causing mitochondria to hyperfuse by Drp1 repression boosts mitochondrial function only in larger cells within diploid proliferating populations [36,156]. It has also been proposed from quantitative theoretical analyses that other factors like cell cycle can also potentially influence the scaling of mitochondria content/function and cell size [157].

The increase in mitochondrial content and cellular mass is molecularly coupled by the mTOR pathway that sustains the cellular anabolic activities related to cell growth [24] and supports stem cell differentiation of various lineages [158]. The energy demanding process of protein translation, regulated by the mTOR pathway, has been proposed to be dependent on mitochondrial energy [159,160], while the mTOR pathway also contributes to the maintenance of mitochondrial content [161]. Therefore, feedback between mitochondrial content and mTOR-driven protein translation has been proposed, where the quantitative aspects of the feedback loop remain an open question. Such a feedback loop could be potentially at play between mTOR and the mitochondrial fission protein Drp1 in the following way. Repression of ribosomal activity with mTOR inhibition represses Drp1 driven mitochondrial fission through the fission regulator MTFP1 [162]. Whereas, repression of Drp1 has been noted to be causatively or correlatively linked to elevated expression of ribosomal genes in proliferating cells, including stem cells [128,133,163]. However, in differentiated cardiac and muscle tissues, Drp1 repression reduces the expression of ribosomal genes and protein synthesis [164,165]. This difference in the impact of mitochondrial fission adds to the distinction in mitochondrial behaviour between proliferating and differentiated cells. The two other regulatory circuitries for cell size control, namely the Hippo kinase pathway and Myc, also have been shown to crosstalk with mitochondrial shape and/or mitochondrial content [22,166–168]. Self-renewing stem cells are usually smaller in size than their differentiated counterparts in the majority of the lineages, indicating the increase in cell size happens along the time line of differentiation [19]. Therefore, we hypothesize that the reported quantitative allometric scaling of mitochondrial content/functionality with cell size, through alteration of mitochondrial shape, is established during differentiation of stem cells (hypothesis III in figure 1).

## 5. Proposed hypotheses testing

Studying the significance of maintaining and modulating a particular mitochondrial shape in a given cell is an active field of research and has been covered in several reviews [98,99,169]. The standard approach generally includes three steps: (a) defining the mitochondrial shape; (b) establishing the correlation of mitochondrial shape with levels/activity of mitochondrial fission and fusion proteins; (c) studying the mechanism of impact of genetic or pharmacological manipulation of the relevant mitochondrial fission or fusion proteins on mitochondrial structure–function and the relevant cell physiology. The majority of the studies on the elucidation of the significance on mitochondrial shape change in stem cell differentiation compare mitochondrial shapes between the stem cells (start point) and their differentiated counterparts (end point). Therefore, some key questions remain open about the timeline of various events during the course of stem cell differentiation. For example, (a) when and how does the mitochondrial shape change happen; (b) when and how the mitochondrial fission and fusion processes are connected to other concomitant mitochondrial changes (ex: increase in mt-DNA copy number) and cellular changes (ex: increase in cell size); (c) the exact functional significance of such mitochondrial shape change. The altered mitochondrial shape and content observed in differentiated cells, their potential interdependence and connection to cell size may be achieved during cell cycle exit, during the process of differentiation or post-differentiation, or in any possible combinations during the course of differentiation (figure 1). Establishment of such a timeline would then allow investigation of the functional significance of such alterations in stem cell differentiation using conditional gene manipulation strategies. The three independent hypotheses laid out in the previous three sections can be tested in a relevant stem cell model along the time course of cell cycle exit, active differentiation and after completion of differentiation. The relevant mitochondrial properties, namely mitochondrial shape metrics, mitochondrial fission–fusion rates, mitochondrial content metrics, and cell size metrics, can be measured in single cells with quantitative approaches discussed in the relevant sections. Thereafter, quantitative correlation and mechanistic causation can be established between parameters in the differentiation time line.

In this review, we have covered relevant findings from mammalian cells as well as yeast models, where the latter has proved to be extremely important in understanding fundamental and general concepts of mitochondrial shape. Therefore, the concepts discussed here may also apply to the fascinating diversity of mitochondrial shape and behaviour existing in other taxa like plants, *Chlamydomonas, Planaria,* and others.

## Data Availability

This article has no additional data.
